# Supplementary material for: CUL2LRR1, TRAIP and p97 control CMG helicase disassembly in the mammalian cell cycle
Source: EMBO Rep. 2021 Feb 15;22(3):e52164. doi: 10.15252/embr.202052164 (PMC7926238; doi:10.15252/embr.202052164)
Supplement: Supplementary file 1 — Appendix [file EMBR-22-e52164-s001.pdf]

Table of Contents

Appendix Figure S1	Page 2
Appendix Figure S2	Page 3
Appendix Figure S3	Page 4
Appendix Table S1	Page 5

A

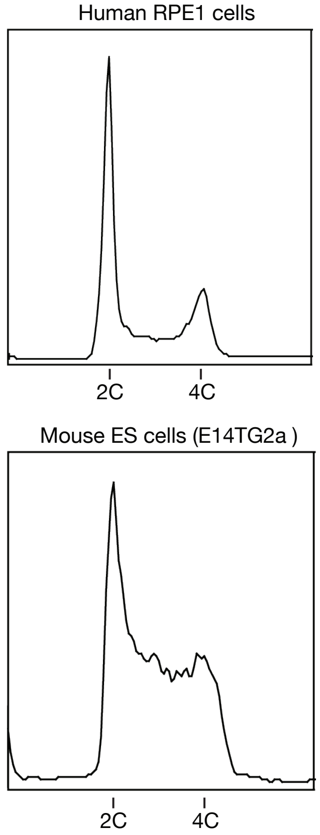

B

	S-phase cells (% EdU +ve)
**Human RPE1**	37%, n= 371
**mES (E14TG2a)**	72%, n= 451

Comparison of the proportion of S-phase cells in asynchronous populations of diploid E14TG2a mouse ES cells and human RPE1 cells.
(**A**) Flow cytometry analysis of DNA content for asynchronously growing E14TG2a mouse ES cells and human RPE1 cells.
(**B**) Proportion of S-phase cells, monitored by quantifying cells that incorporated a pulse of 5-Ethynyl-2’-deoxyuridine (EdU), as described in Materials and Methods.

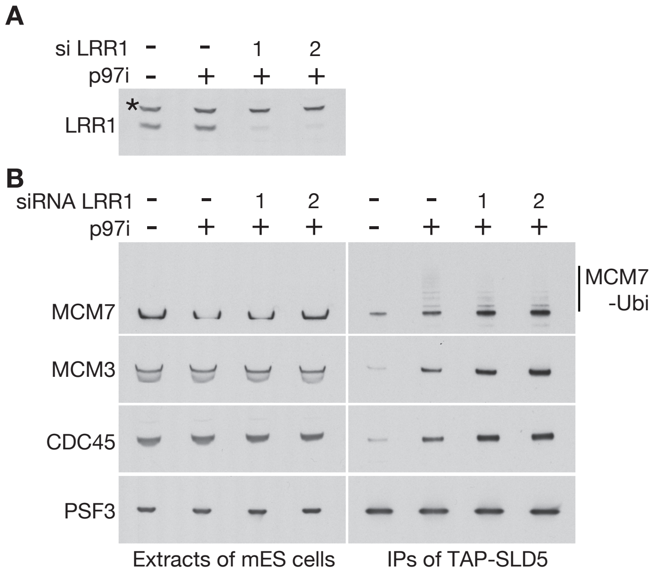

Comparison of two different siRNA for LRR1.
(**A**) TAP-SLD5 cells were treated with the indicated siRNA (see Appendix Table S1 – siRNA2 was used in all other such experiments) and depletion of LRR1 was monitored by immunoblotting. The asterisk indicates a non-specific band.
(**B**) TAP-SLD5 was isolated from cell extracts by immunoprecipitation. The indicated proteins were monitored by immunoblotting

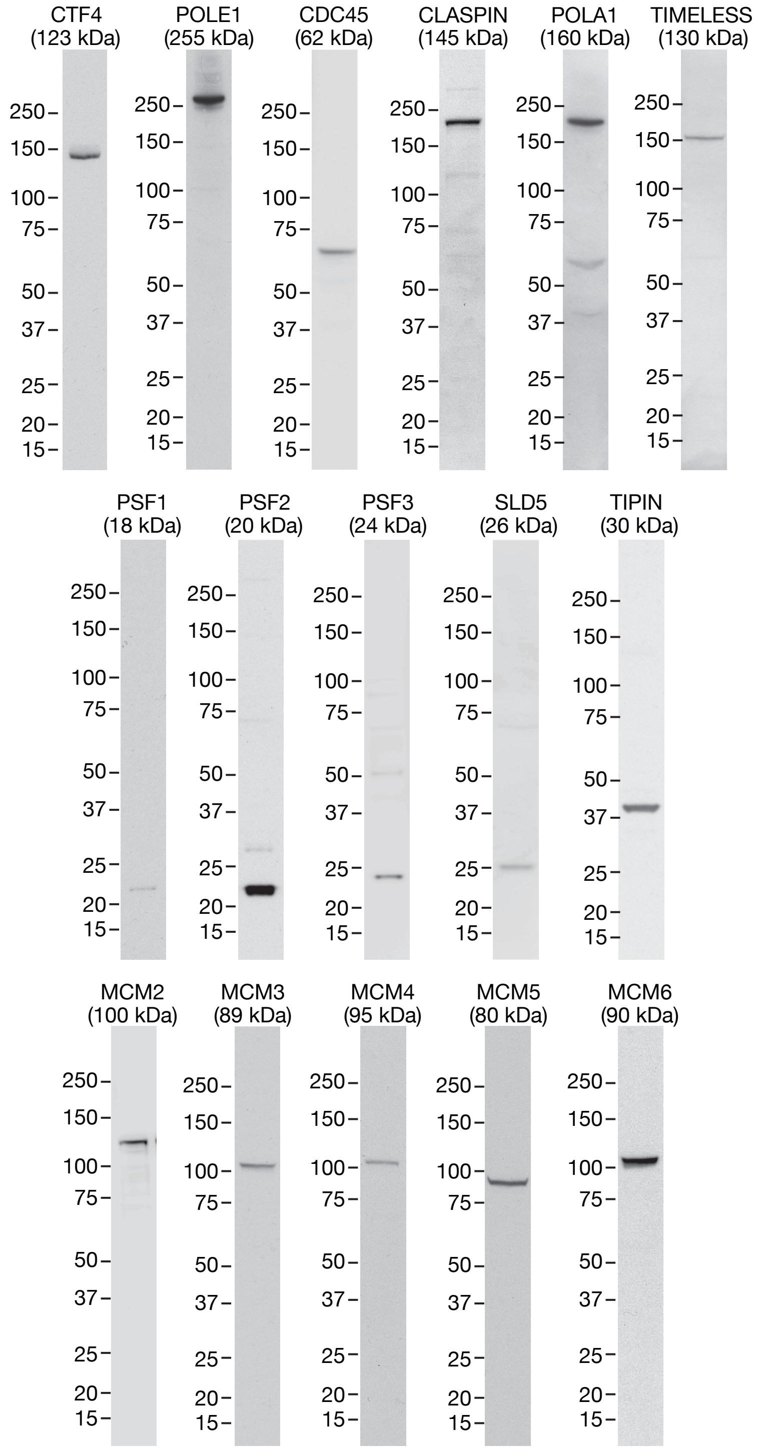

Validation of antibodies produced in this study.
The indicated antibodies were tested via immunoblotting of extracts of mouse ES cells.
The antibody to LRR1 is validated in Figure 3B and Figure EV2C, whereas the antibodies to TRAIP are confirmed in Figure 5C and Figure EV4E. See Appendix Table S1 for more details.

Appendix Table S1

Reagents and resources used in this study.

**REAGENT or RESOURCE**	SOURCE	IDENTIFIER
**Antibodies**		
MCM2 [antigen 1-222 mouse]<br>Use 1 in 5,000 for immunoblotting	MRC PPU Reagents and Services	DU24572
MCM3 [antigen 1-222 mouse]<br>Use 1 in 1,000 for immunoblotting	MRC PPU Reagents and Services	DU51847
MCM4 [antigen 1-222 mouse]<br>Use 1 in 3,000 for immunoblotting	MRC PPU Reagents and Services	DU51810
MCM5 [antigen 1-222 mouse]<br>Use 1 in 3,000 for immunoblotting	MRC PPU Reagents and Services	DU51792
MCM6 [antigen 1-222 mouse]<br>Use 1 in 3,000 for immunoblotting	MRC PPU Reagents and Services	DU51793
MCM7 [antigen 1-719 human]<br>Use 1 in 500 for immunoblotting	Santa Cruz Biotechnology	sc-9966
PSF1 [antigen 1-196 mouse]<br>Use 1 in 3,000 for immunoblotting	MRC PPU Reagents and Services	DU51938
PSF2 [antigen 1-185 mouse]<br>Use 1 in 3,000 for immunoblotting	MRC PPU Reagents and Services	DU51832
PSF3 [antigen 1-216 mouse]<br>Use 1 in 1,000 for immunoblotting	MRC PPU Reagents and Services	DU24601
SLD5 [antigen 1-223 mouse]<br>Use 1 in 2,000 for immunoblotting	MRC PPU Reagents and Services	DU24572
CDC45 [antigen 1-222 mouse]<br>Use 1 in 1,000 for immunoblotting	MRC PPU Reagents and Services	DU35753
CTF4 [antigen 781-1001 mouse]<br>Use 1 in 1,000 for immunoblotting<br>Use 1 in 100 for immunofluorescence	MRC PPU Reagents and Services	DU56612
CLASPIN [antigen 1-222 mouse]<br>Use 1 in 1,000 for immunoblotting<br>Use 1 in 100 for immunofluorescence	MRC PPU Reagents and Services	DU56412
TIMELESS [antigen 1-222 mouse]<br>Use 1 in 500 for immunoblotting	MRC PPU Reagents and Services	DU56427

TIPIN [antigen 1-222 mouse]<br>Use 1 in 3,000 for immunoblotting	MRC PPU Reagents and Services	DU56426
POLA1 [antigen 222-443 mouse]<br>Use 1 in 3,000 for immunoblotting	MRC PPU Reagents and Services	DU47647
POLE1 [antigen 1-222 mouse]<br>Use 1 in 1,000 for immunoblotting<br>Use 1 in 100 for immunofluorescence	MRC PPU Reagents and Services	DU27959
LRR1 [antigen 1-160 mouse]<br>Use 1 in 500 for immunoblotting	MRC PPU Reagents and Services	DU51811
TRAIP [antigen 311-470 mouse]<br>Use 1 in 2,000 for immunoblotting	MRC PPU Reagents and Services	DU62040
TRAIP [antigen 1-204 human]<br>Use 1 in 1,000 for immunoblotting	Protein-tech	10332-1-AP
VHL [antigen 1-181 mouse]<br>Use 1 in 1,000 for immunoblotting	Santa Cruz Biotechnology	sc-55506
HP1 [antigen 1-191 human]<br>Use 1 in 100 for immunofluorescence	Santa Cruz Biotechnology	sc-515341
anti-sheep IgG HRP [from donkey]<br>Use 1 in 10,000 for immunoblotting	Sigma-Aldrich	A3415
anti-mouse IgG HRP [from goat]<br>Use 1 in 10,000 for immunoblotting	Sigma-Aldrich	A4416
anti-rabbit IgG HRP [from goat]<br>Use 1 in 10,000 for immunoblotting	Sigma-Aldrich	A6667
anti-sheep IgG Alexa Fluor 594 [from donkey]<br>Use 1 in 1,000 for immunofluorescence	Invitrogen	A11016
anti-mouse IgG Alexa Fluor 594 [from donkey]<br>Use 1 in 1,000 for immunofluorescence	Invitrogen	A21203
**Chemicals, Peptides, and Recombinant Proteins**		
LIF	MRC PPU Reagents and Services	DU1715
Ubiquitin PrG	MRC PPU Reagents and Services	DU49003
USP2	MRC PPU Reagents and Services	DU32888
CB-5083	Selleckchem	S8101
MLN-4924	Activebiochem	A1139
MMC	Duchefa Biochemie	M0133

S-Trityl-L-cysteine (STLC)	Sigma	164739
Thymidine	Sigma	T9250
**Experimental Models: Organisms/Strains**		
E14tg2A mouse ES cells		N/A
E14tg2A *TAP-SLD5*	This study	N/A
E14tg2A *GFP-SLD5*	This study	N/A
E14tg2A *TRAIPΔ*	This study	N/A
E14tg2A *GFP-SLD5 TRAIPΔ ROSA26::TRAIP*	This study	N/A
E14tg2A GFP-SLD5 TRAIPΔ *ROSA26::TRAIP^{R}* (W37F RING mutant of TRAIP)	This study	N/A
E14tg2A *GFP-SLD5 TRAIPΔ ROSA26::TRAIP^{PIP}* (Q461A L464A PIP mutant of TRAIP)	This study	N/A
E14tg2A *GFP-SLD5 mCHERRY-Histone H2B*	This study	N/A
E14tg2A *GFP-SLD5 mCHERRY-Histone H2B TRAIPΔ*	This study	N/A
E14tg2A *GFP-SLD5 mCHERRY-PCNA*	This study	N/A
hTERT RPE-1 human retinal pigment epithelial cells		N/A
**Oligonucleotides**		
SLD5 gRNA1_oligo 1 caccgCTCCGTCATCGTTTCTCAAC	This study	N/A
SLD5 gRNA1_oligo 2 aaacGTTGAGAAACGATGACGGAGc	This study	N/A
SLD5 gRNA2_oligo 1 caccgTCCGTCATCGTTTCTCAACC	This study	N/A
SLD5 gRNA2_oligo 2 aaacGGTTGAGAAACGATGACGGAc	This study	N/A
SLD5 gRNA3_oligo 1 caccgACGGAGGTTCTGGATCTCCA	This study	N/A
SLD5 gRNA3_oligo 2 aaacTGGAGATCCAGAACCTCCGTc	This study	N/A
TRAIP gRNA1_oligo 1 caccgCAGAGCGCGGATAGGCATGA	This study	N/A
TRAIP gRNA1_oligo 2 aaacTCATGCCTATCCGCGCTCTGc	This study	N/A
TRAIP gRNA2_oligo 1 caccgCTTCGATCACTCCCGTGACG	This study	N/A
TRAIP gRNA2_oligo 2 aaacCGTCACGGGAGTGATCGAAGc	This study	N/A
ROSA26 gRNA1_oligo 1 caccgACTGGAGTTGCAGATCACG	This study	N/A
ROSA26 gRNA1_oligo 2 aaacCGTGATCTGCAACTCCAGTc	This study	N/A

SLD5 PCR_oligo 1 TTCTTGCAAGTAAGTGGGAGAAGC	This study	N/A
SLD5 PCR_oligo 2 CAATGAGCTCTGCAGGAGTTAGGAC	This study	N/A
TRAIP PCR_oligo 1 GTTATTCCTTCCGGTTCACTACCGG	This study	N/A
TRAIP PCR_oligo 2 CCGACACCCCCATAGCGTCTAAGTG	This study	N/A
ROSA26 PCR_oligo 1 CCTGCAGGACAACGCCCACACAC	This study	N/A
ROSA26 PCR_oligo_2 TGAGGACCGCCCTGGGCCTGGG	This study	N/A
LRR1 siRNA # 1 CAACAUUUGACUCGAAUUU	Horizon Discovery	J-057816-09
LRR1 siRNA # 2 CGACUACAAUGAACCUACA	Horizon Discovery	J-057816-10
VHL siRNA pool	Horizon Discovery	L-040755-00
**Recombinant DNA (Plasmids)**		
pX335 [expression vector for gRNA and Cas9-D10A 'nickase']	Pyzocha et al (2014)	Addgene
pKN7 [expression vector for gRNA and Puromycin-resistance gene]	MRC PPU Reagents and Services	DU70250
pKN101 [expression vector for gRNA, Cas9-D10A 'nickase' and Puromycin-resistance gene]	MRC PPU Reagents and Services	DU70263
pKN42 [gRNA1 SLD5; based on pX335]	MRC PPU Reagents and Services	DU70251
pKN150 [gRNA2 SLD5; based on pKN101]	MRC PPU Reagents and Services	DU70252
pKN43 [gRNA3 SLD5; based on pKN7]	MRC PPU Reagents and Services	DU70253
pFV79 [gRNA1 TRAIP; based on pX335]	MRC PPU Reagents and Services	DU70254
pFV76 [gRNA2 TRAIP; based on pKN7]	MRC PPU Reagents and Services	DU70255
pKN88 [gRNA ROSA26; based on pKN101]	MRC PPU Reagents and Services	DU70256

pKN161 [donor vector for TAP-SLD5]	MRC PPU Reagents and Services	DU70257
pRF011 [donor vector for GFP-SLD5]	MRC PPU Reagents and Services	DU70258
pRF014 [donor vector for TRAIP WT]	MRC PPU Reagents and Services	DU70259
pRF027 [donor vector for $TRAIP^{R}$]	MRC PPU Reagents and Services	DU70260
pRF039 [donor vector for $TRAIP^{PIP}$]	MRC PPU Reagents and Services	DU70261
pRF010 [donor vector for mCHERRY-H2B]	MRC PPU Reagents and Services	DU70262
pcDNA3.1-mCherry-PCNA [donor vector for mCHERRY-PCNA]	Masato Kanemaki	N/A
**Software and Algorithms**		
ImageJ	National Institute of Health	https://imagej.nih.gov/ij/
