# Supplementary material for: CUL2LRR1, TRAIP and p97 control CMG helicase disassembly in the mammalian cell cycle
Source: EMBO Rep. 2021 Feb 15;22(3):e52164. doi: 10.15252/embr.202052164 (PMC7926238; doi:10.15252/embr.202052164)
Supplement: Supplementary file 2 — Expanded View Figures PDF [file EMBR-22-e52164-s002.pdf]

Expanded View Figures

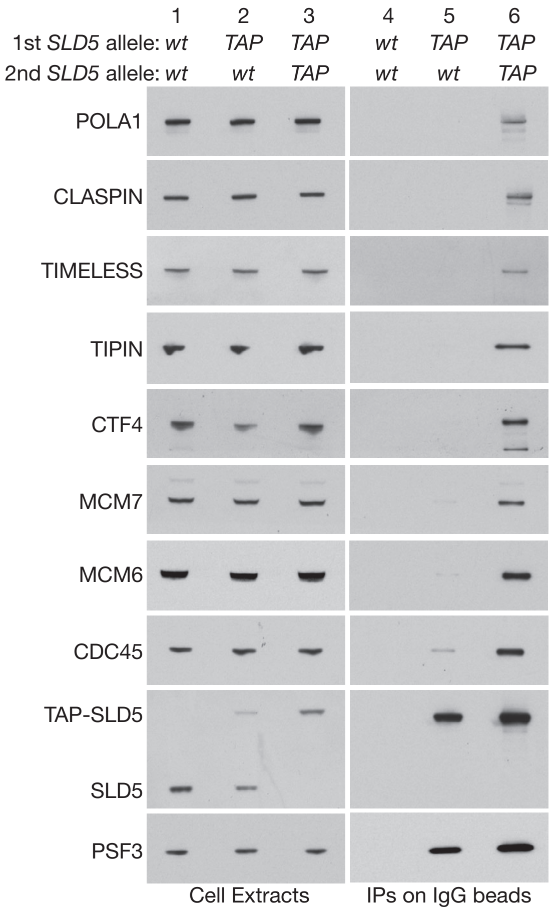

Figure EV1. GINS co-purifies with a range of replisome components from homozygous *TAP-SLD5* mouse ES cells.

Cell extracts from mouse ES lines with the indicated genotypes were incubated with IgG beads. The isolated proteins were analysed by immunoblotting in order to monitor the factors that co-purified with *TAP-SLD5*. Note that isolation of the CMG was more effective from homozygous *TAP-SLD5* / *TAP-SLD5* cells than from the equivalent heterozygote

 © 2021 The Authors

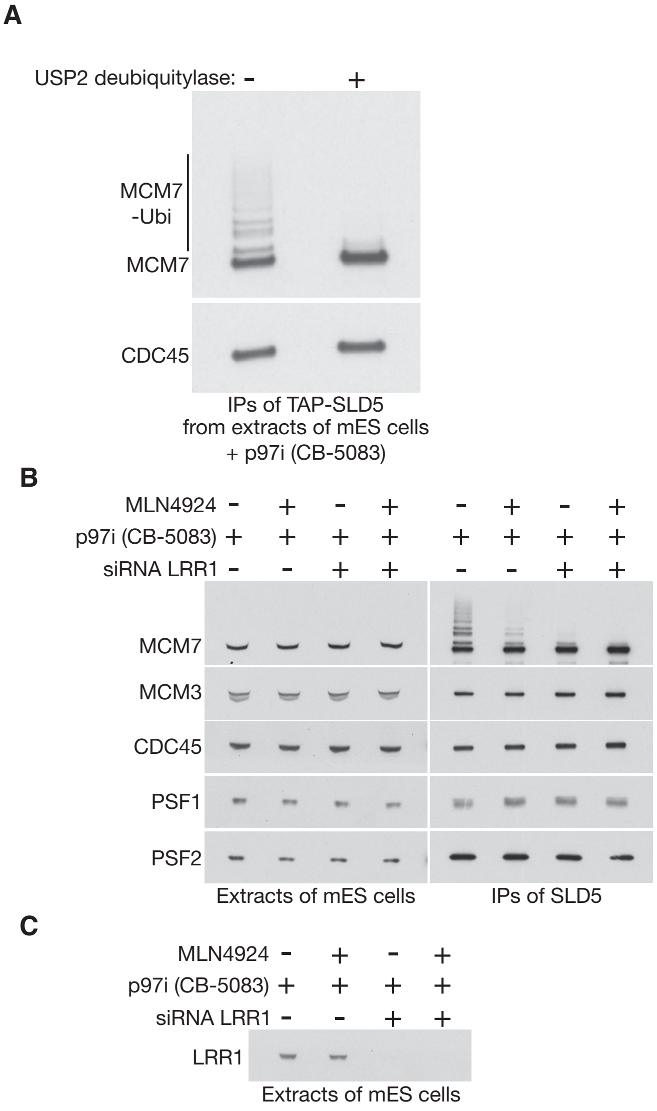

Figure EV2. Ubiquitylation of CMG-MCM7 by CUL2^{LRR1}.

A Ubiquitylated CMG was isolated from extracts of mouse ES cells treated with 5 μM CB-5083, by immunoprecipitation of TAP-SLD5. Subsequently, the immunoprecipitates were treated with the USP2 deubiquitylase as indicated, confirming that the observed modifications of CMG-MCM7 corresponded to ubiquitylation.

B *TAP-SLD5* cells were treated as indicated and TAP-SLD5 was then isolated from cell extracts by immunoprecipitation. The indicated proteins were monitored by immunoblotting.

C Depletion of LRR1 was monitored by immunoblotting for the experiment in (B).

© 2021 The Authors

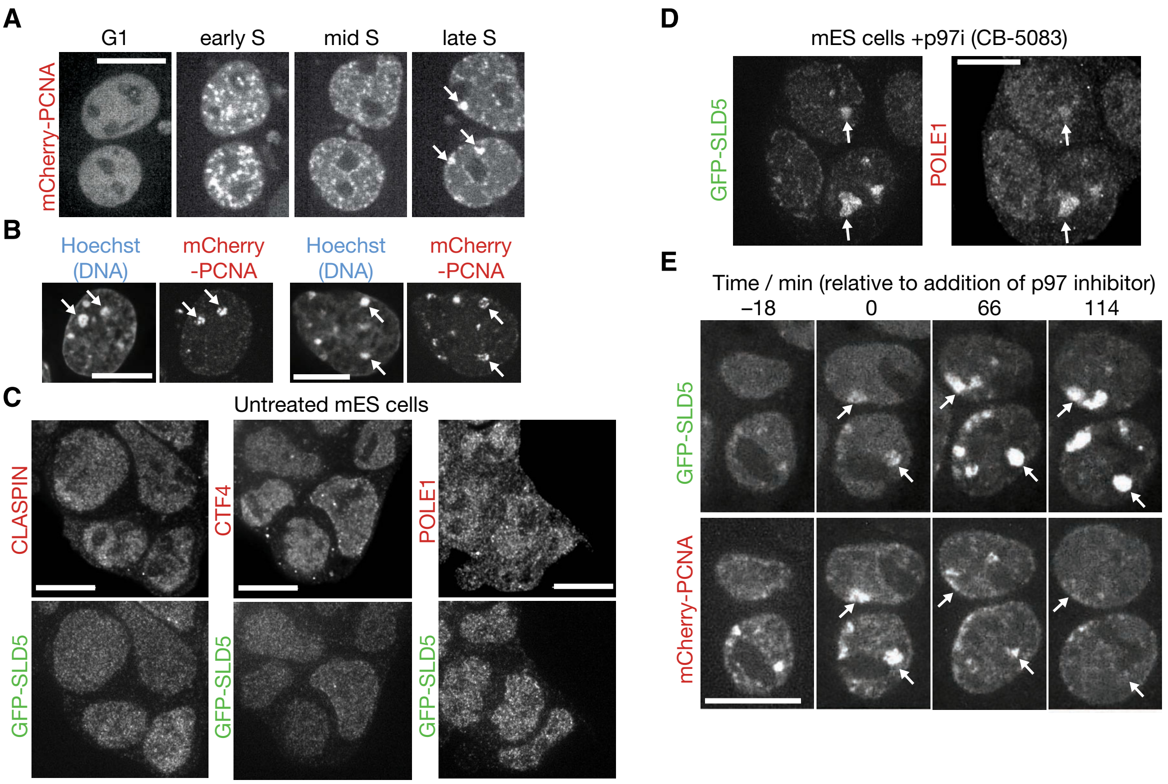

Figure EV3. Accumulation of replisome components on chromatin in response to p97 inhibition.

- A Time-lapse analysis of mCherry-PCNA as mouse ES cells enter and progress through S-phase (arrows denote large patches that are seen in late S-phase).
- B Cells expressing mCherry-PCNA were fixed and stained with Hoechst, revealing that large mCherry-PCNA patches in late S-phase correspond to replication of constitutive heterochromatin (examples marked by arrows). In 94% of cells with large PCNA patches, these co-localised with condensed DNA, n = 53.
- C The indicated replisome factors were analysed by immunofluorescence in untreated mouse ES cells expressing GFP-SLD5.
- D Similar experiment to that in (C), using cells treated with 5 μM CB-5083. The arrows indicate the co-localisation of POLE1 and GFP-SLD5 on heterochromatic patches (88% of cells, n = 49).
- E Time-lapse analysis of mouse ES cells expressing GFP-SLD5 and mCherry-PCNA, following treatment with the p97 inhibitor CB-5083. The arrows illustrate the persistence of GFP-SLD5 on heterochromatic patches after the disappearance of mCherry-PCNA (100% of cells, n = 77).

Data information: Scale bars in all panels correspond to 10 μm.

Figure EV4. TRAIP$^{-/-}$ mouse ES cells are viable but are sensitive to DNA damaging agents that induce inter-strand DNA crosslinks. ▶

- A Procedure for creating small deletion at 5′ end of first exon of the *TRAIP* gene in mouse ES cells.
- B Location of gRNAs that were used to target the Cas9-D10A “nickase” to the *TRAIP* locus. The panel also indicates the location of two PCR oligos that were used subsequently to check the integrity of the targeted region.
- C PCR analysis of genomic DNA from cells transfected with DNA expressing Cas9-D10A and the indicated gRNA(s) from (B).
- D DNA sequence analysis of the targeted region from control cells and two clones exposed to Cas9-D10A in the presence of gRNAs 1 + 2 (PAM = Protospacer Adjacent Motif). See Materials and Methods for further details.
- E Immunoblots of cell extracts from control cells and *TRAIPΔ* clones, using the indicated anti-TRAIP antibodies. Asterisks indicate non-specific bands.
- F (i) Flow cytometry analysis of DNA content for asynchronously growing wild-type and TRAIP$^{-/-}$ mouse ES cells. (ii) Doubling times were calculated as described in Materials and Methods.
- G Procedure for expressing wild-type or mutated TRAIP at the *ROSA26* locus in TRAIP$^{-/-}$ cells.
- H Cells with the indicated genotypes were grown for 24 h in the presence of varying concentrations of the DNA damaging agent Mitomycin C as shown, before continued growth in the absence of drug.

Data Information: In (F), the doubling time data are presented as the mean values of three experiments ± standard deviation.

 © 2021 The Authors

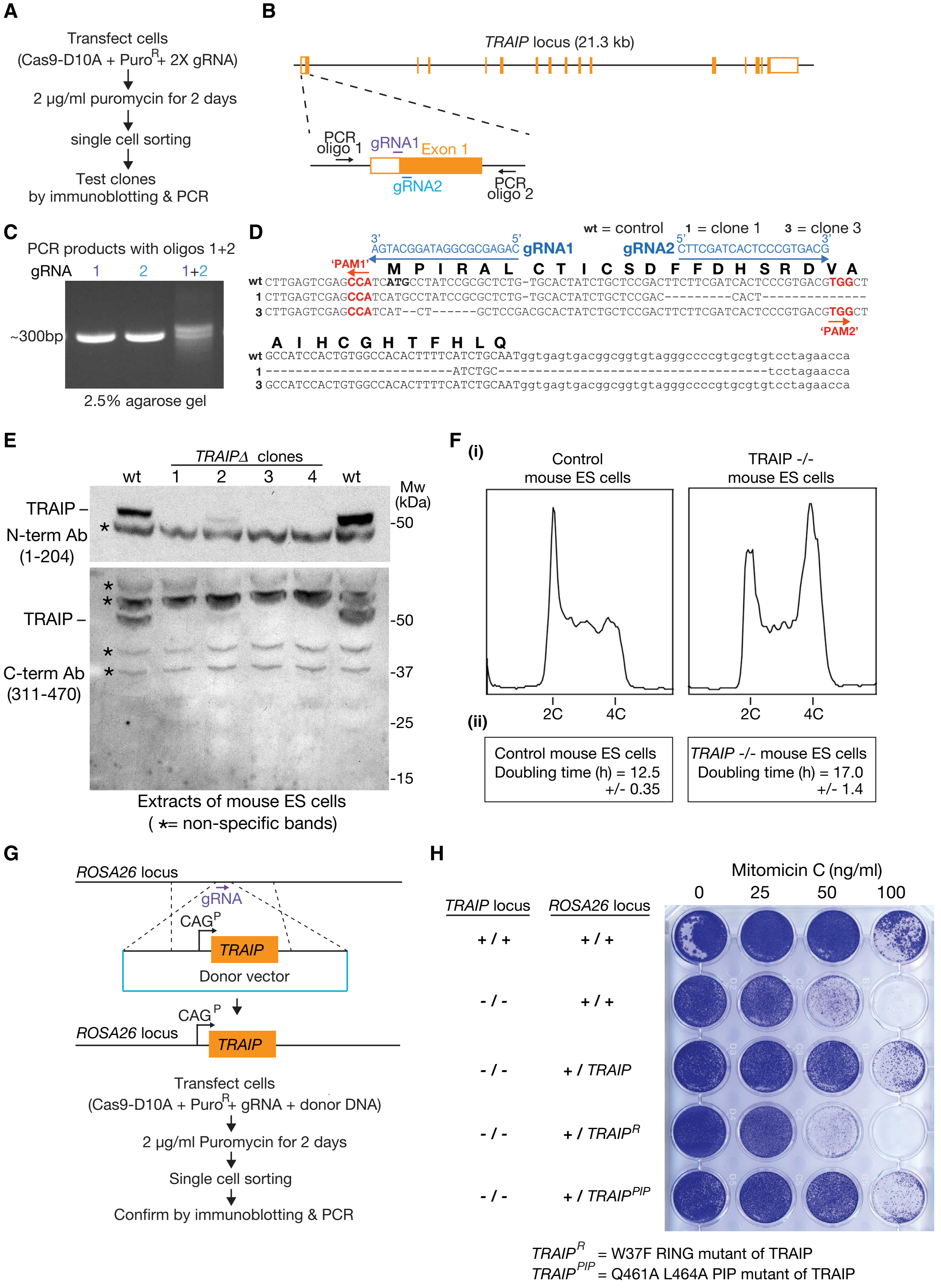
A
Transfect cells
(Cas9-D10A + Puro^R + 2X gRNA)
2 µg/ml puromycin for 2 days
single cell sorting
Test clones
by immunoblotting & PCR
B
TRAIP locus (21.3 kb)
PCR oligo 1
gRNA1
Exon 1
gRNA2
PCR oligo 2
C
PCR products with oligos 1+2
gRNA 1 2 1+2
~300bp
2.5% agarose gel
D
wt = control 1 = clone 1 3 = clone 3
3' AGTACGGATAGGCGCGAGAC 5' gRNA1
gRNA2 5' CTTCGATCACTCCCGTGACG 3'
'PAM1'
M P I R A L C T I C S D F F D H S R D V A
wt CTTGAGTCGAGCCATCATGCCTATCCGCGCTCTG-TGCACTATCTGCTCCGACTTCTTCGATCACTCCCGTGACGTGGCT
1 CTTGAGTCGAGCCATCATGCCTATCCGCGCTCTG-TGCACTATCTGCTCCGAC---------CACT--------------
3 CTTGAGTCGAGCCATCAT--CT------GCTCCGACGCACTATCTGCTCCGACTTCTTCGATCACTCCCGTGACGTGGCT
'PAM2'
A I H C G H T F H L Q
wt GCCATCCACTGTGGCCACACTTTTCATCTGCAATggtgagtgacggcggtgtagggccccgtgcgtgtcctagaacca
1 ------------------------ATCTGC-----------------------------------tcctagaacca
3 GCCATCCACTGTGGCCACACTTTTCATCTGCAATggtgagtgacggcggtgtagggccccgtgcgtgtcctagaacca
E
TRAIPΔ clones
wt 1 2 3 4 wt
Mw (kDa)
TRAIP –
N-term Ab (1-204)
-50
TRAIP –
C-term Ab (311-470)
-50
-37
-25
-15
Extracts of mouse ES cells
(⋆= non-specific bands)
F
(i)
Control mouse ES cells
TRAIP -/- mouse ES cells
2C 4C
2C 4C
(ii)
Control mouse ES cells
Doubling time (h) = 12.5 +/- 0.35
TRAIP -/- mouse ES cells
Doubling time (h) = 17.0 +/- 1.4
G
ROSA26 locus
gRNA
CAG^P
TRAIP
Donor vector
CAG^P
ROSA26 locus
TRAIP
Transfect cells
(Cas9-D10A + Puro^R + gRNA + donor DNA)
2 µg/ml Puromycin for 2 days
Single cell sorting
Confirm by immunoblotting & PCR
H
Mitomicin C (ng/ml)
0 25 50 100
TRAIP locus ROSA26 locus
+ / + + / +
- / - + / +
- / - + / TRAIP
- / - + / TRAIP^R
- / - + / TRAIP^PIP
TRAIP^R = W37F RING mutant of TRAIP
TRAIP^PIP = Q461A L464A PIP mutant of TRAIP

Figure EV4.

Figure EV5. Models for disassembly of the metazoan replisome. The figure summarises data from this manuscript together with previous studies with early embryos of *Caenorhabditis elegans* and extracts of *Xenopus laevis* eggs.

A During DNA replication termination, $CUL2^{LRR1}$ promotes ubiquitylation of CMG-MCM7, leading to disassembly by p97-UFD1-NPL4.

B In cells that lack $CUL2^{LRR1}$ activity, the CMG replisome remains on chromatin after DNA replication termination. Upon entry into mitosis, the TRAIP ubiquitin ligase activates a second pathway of CMG disassembly, dependent upon p97.

C Previous work showed that TRAIP is required in human cells to process sites of incomplete DNA replication during mitosis, by a process of Mitotic DNA Synthesis or "MiDAS" (Sonneville *et al*, 2019). It is likely that the first step in this pathway is TRAIP-dependent CMG disassembly upon entry into mitosis.

 © 2021 The Authors

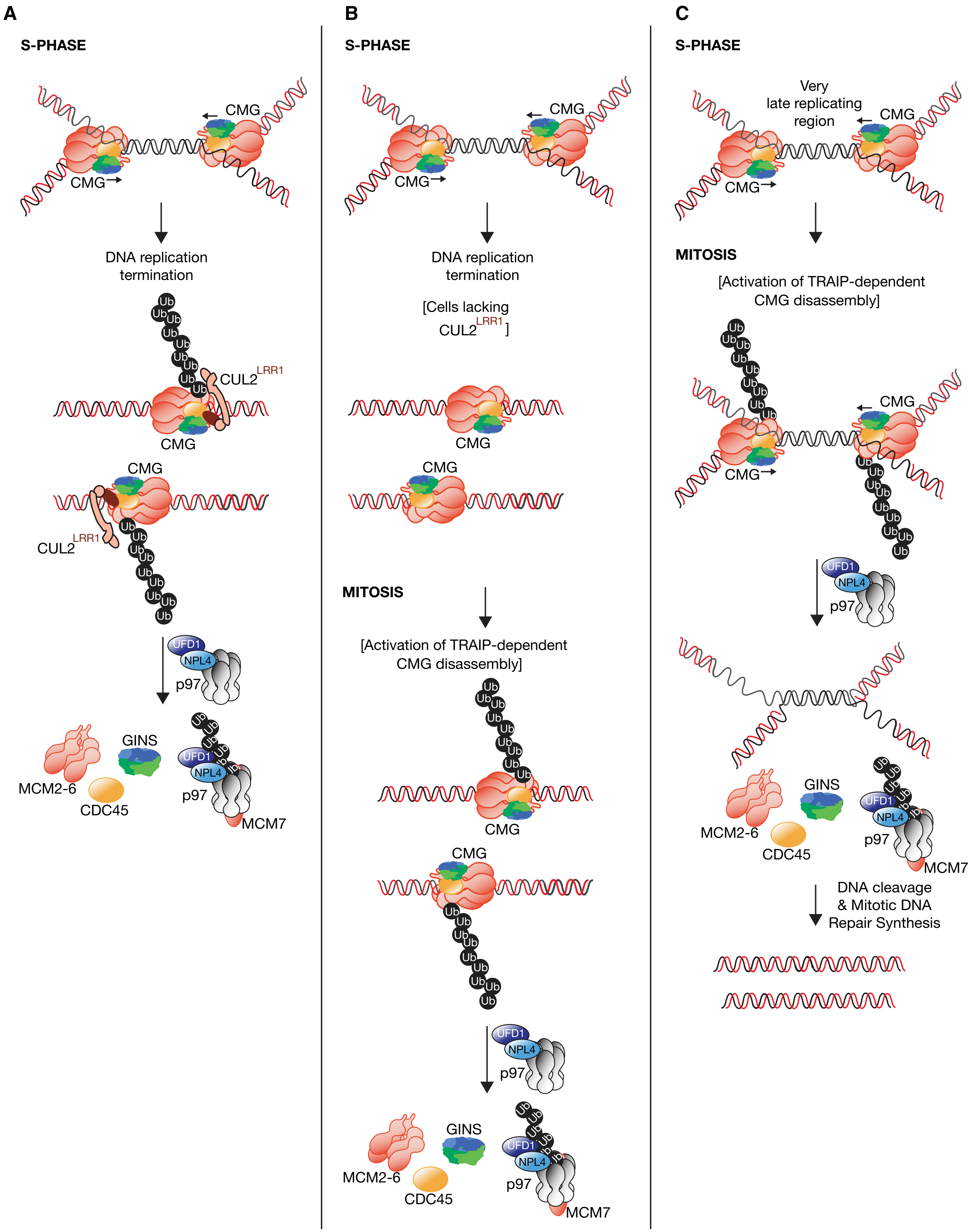
A
S-PHASE
CMG
CMG
DNA replication
termination
CUL2LRR1
CMG
CMG
CUL2LRR1
UFD1
NPL4
p97
GINS
MCM2-6
CDC45
p97
MCM7
B
S-PHASE
CMG
CMG
DNA replication
termination
[Cells lacking
CUL2LRR1]
CMG
CMG
MITOSIS
[Activation of TRAIP-dependent
CMG disassembly]
CMG
CMG
UFD1
NPL4
p97
GINS
MCM2-6
CDC45
p97
MCM7
C
S-PHASE
Very
late replicating
region
CMG
CMG
MITOSIS
[Activation of TRAIP-dependent
CMG disassembly]
CMG
CMG
UFD1
NPL4
p97
GINS
MCM2-6
CDC45
p97
MCM7
DNA cleavage
& Mitotic DNA
Repair Synthesis

Figure EV5.
